# Osteogenic Inhibition in Multiple Myeloma

**Published:** 2013-08-24

**Authors:** Hussain Habibi, Saeid Abroun, Abbas Hajifathali, Masoud Soleimani, Saeid Kaviani, Nasim Kalantari, Susan Eslahchi

**Affiliations:** 1 Department of Hematology, School of Medical Sciences, Tarbiat Modares University, Tehran, Iran; 2 Department of Hematology, Taleghany Hospital, Shahid Beheshti Medical University, Tehran, Iran

**Keywords:** Multiple Myeloma, Osteoblast, Sclerostin, *SOST*, Cord Blood Stem Cells

## Abstract

**Objective:**

Multiple myeloma (MM) is a plasma cell malignancy where plasma cells are increased in the bone marrow (BM) and usually do not enter peripheral blood, but produce harmful factors creating problems in these patients (e.g. malignant plasma cells over activate osteoclasts and inhibit osteoblasts with factors like RANKL and DKK). These factors are a main cause of bone lesion in MM patients. Recently *SOST* gene which responsible to encodes the sclerostin protein was identify. This protein specifically inhibits Wnt signaling in osteoblasts (inhibition of osteoblast differentiation and proliferation) and decrease bone formation and can also cause bone lesion in MM patients.

**Materials and Methods:**

In this experimental study, human myeloma cell lines (U266 b1) were purchased from Pasteur Institute of Iran. Samples consisted of BM aspirates from the iliac crest of MM patients. BM with more than 70% plasma cell were selected for our study (6 patients) and one healthy donor. RNA extraction was done with Qiagen kit. was undertaken on mRNA of samples and cell lines. Also we purchased unrestricted somatic stem cells from Bonyakhte Company to evaluate the effect of soluble factors from myeloma cell lines on osteogenic differentiation medium.

**Results:**

Our results showed that *SOST* is expressed significantly in primary myeloma cells derived from MM patients and myeloma cell lines. In other words, patients with more bone problems, express *SOST* in their plasma cells at a higher level. In addition, myeloma cells inhibit osteoblast differentiation in progenitor cells from umbilical cord blood stem cell (UCSC) in osteogenic inducing medium.

**Conclusion:**

There are many osteoblast maturation inhibitory factors such as DKK, Sfrp and Sclerostin that inhibit maturation of osteoblast in bone. Among osteoblast inhibitory agents (DKK, Sfrp, Sclerostin) sclerostin has the highest specificity and therefore will have less side effect versus non-specific inhibitory agents. Our results also show that based on *SOST* expression in MM, there is a potential to inhibit sclerostin with antibody or alternative methods and prevent bone lesion in MM patients with the least side effect.

## Introduction

tiple myeloma (MM) is a plasma cell malignancy where plasma cells reside in bone marrow (BM) and usually do not enter peripheral blood (1). Bone diseases are usually the hallmark of multiple myeloma and occur more frequently in patients with hypercalcemia, bone fractures, bone pain influencing the life quality of MM patients (2-4). Establishment of myeloma cells in BM, leading to bone resorption, follows by bone pain, bone fracture, hypercalcemia and anemia in MM patients (5, 6). Clonal expansion of myeloma plasma cells (CD138+ ) increases the production of monoclonal antibody which is a diagnostic sign of this disease. Light chain monoclonal antibody and hypercalcemia are causes of renal failure which is a clinical hallmark of MM (7). Inhibition of osteoclast and increase in osteoblast function can treat these problems in MM patients (8). Osteoclastogenesis is regulated by a signaling pathway like receptor activator of nuclear factor-kB (RANK) that are expressed on the surface of osteoclasts lineage and RANK ligand (RANKL) that are secreted by BM stromal cells (BMSCs). Osteoblastogenesis include proliferation and differentiation of BMSCs into functional osteoblast that involve factors such as Cbfa1/Runx2, which directly influence the expression of osteoblast markers such as collagen type I, osteopontin, bone sialoprotein(6, 9). Wnt is a cysteine-rich glycoprotein that activates cell surface specific receptor-mediated signaling to control gene expression, differentiation, proliferation, and migration. Wnt is an essential factor for organogenesis, embryogenesis postnatal development and regeneration of adult tissues consisting of lymphocytes, skin, colon, hair follicles, and bone (10, 11). The role of Wnt signaling in bone formation was established by Giuliani et al. (8). Their studies have confirmed the role of Wnt signaling in bone formation. There are two basic therapeutic targets for enhancing bone formation by the Wnt signaling: adding Wnt agonists and blocking inhibitors of Wnt with antibody or an alternative procedure like epigenetic ways. Recombinant Wnt is unreasonable and difficult because it is a glycoprotein and only its palmitoylate form is functional. The other strategy is inhibition of Wnt antagonists which is a more plausible approach. In this way secreted inhibitors of Wnt signaling can be neutralized with antibodies (9, 12). Recently *SOST* gene which located on chromosome 17 and responsible to encodes the sclerostin protein was identify. This protein specifically inhibits Wnt signaling in Osteoblasts (inhibition of osteoblast differentiation and proliferation) and decrease bone formation (13-16). Genetic studies discovered the genetic cause of Sclerosteosis and van Buchem disease, characterized by enhancement in bone mass (14). *SOST* plays a role in the pathogenesis of van Buchem disease and Sclerosteosis in both of which sclerostin does not have functional activity and so an increase in bone mass without any tumor is observed since sclerostin is expressed in MM and Osteogenic Inhibition the bone and is crucial for osteoblast maturation (14, 15). MM patients show bone mass decline and reduction in osteoblast formation (5, 17). One way to prevent bone lesion in MM patient is osteoblast activation by neutralization of Wnt inhibitors consisting of DKK, Sfrp and WISE. However,they have many side effects because Wnt pathway is involved in organogenesis, embryogenesis, postnatal development and regeneration of adult tissues including lymphocytes, skin, colon, hair follicles, and bone (14, 18, 19).

Another approach is to inhibit sclerostin activation as a suitable therapeutic strategy. This study examined *SOST* expression in plasma of MM patients and its effect on UCSC differentiation to osteoblast in osteogenic medium MM cells secret sclerostin which it can inhibit osteoblast differentiation. 

## Materials and Methods

### Cell lines and conditioned media

Myeloma cell line U266 was purchased from Iran Pasture Institute and it was checked for expression of CD138, CD38 and CD19. Also HEK T293 and K562 cell lines were used as control (Table 1).

**Table 1:** Characteristic of cells line


Cell	Line ATCC Number	No

**U266**	TIB-196	Myeloma
**K562**	CCL-243	CML
**HEK T 293**	CRL-1573	Human embryonic kidney


### UCSCs

tal study, we purchased UCSC from Bonyakhte Company (Iran) and for assurance evaluated specific markers of these cells. All of these cells expressed CD105 and were negative for CD49, CD26 and CD146. These cells were used to evaluate the effect of soluble factors from myeloma cells line culture in RPMI medium on osteogenic differentiation. Then UCSC were cultured in DMEM medium (Sigma, USA) with 12% fast blood suger (FBS, Sigma, USA), in 80% of cell confluence DMEM medium change with osteogenic medium (Low glucose DMEM, 10 mM β-glycerophosphate, 50 µmol ascorbic acid, 0.1 µM dexamethasone) (20), and then added 1cc conditioned media of myeloma cell culture to osteogenic medium consisting of UCSC. Whereas sclerostin is a secretory protein that can be secreted into conditioned media from myeloma cells line and when this conditioned media is added to osteogenic medium consisting of UCSC, it can inhibit osteogenic differentiation of UCSC to Osteoblast. After 7, 14 and 21 days RNA extraction was done and expression of Runx2 with RT-PCR as the specific marker for osteoblast differentiation was evaluated. In this study we used cultured UCSC in osteogenic medium without myeloma cells line conditioned media as positive control and UCSC in DMEM with 12% FBS as negative control.

### Sampling from MM patients

We collected BM patients with informed written consent according to the Medical Ethics Committee of Tarbiat Modares University. BM of patients were confirmed for presence of malignant plasma cells by a hemato-pathologist, and also were analyzed for CD19, CD38 and CD138 expression by flow cytometry. 

BM with more than 70% plasma cell was selected for our study (6 patients) along with one healthy donor as control. BM mononuclear cells were isolated with Ficol-Paque.

### RNA extraction and cDNA synthesis for plasma cells, myeloma cell line and differentiated cells

RNA extraction was undertaken with QIAGEN kit (USA) and for assurance quality of RNA, with Biophotometer measured concentration and their purity at OD in 260 and 280. Afterwards for cDNA synthesis we used QIAGEN kit (Quantitect® Reverse Transcription).

### Primers

The following primers were used to amplify human *SOST* fragment F: 5´ ACACAGCCTTCCGTGTAGT3´, R: 5´TCGGACACGTCTTTGGTCT3´ (product size 186 bp) and Human Runx2 fragment F: 5´ GCCTTCAAGGTGGTAGCCC 3´, R: 5´CGTTACCCGCCATGACAGTA3´ (product size 66 bp) and Human β2microglobin fragment F: 5´CCAGCAGAGAATGGAAAGTC3´, R: 5´GATGCTGCTTACATGTCTCG3´ (product size 269bp). PCR was then implemented at 25 µl reaction in 0.2 ml microtubes with the conditions of annealing and extension temperatures of 60˚C and 71˚C and 35 cycles.

### Alizarin red stain

This stain was done on differentiation culture to confirm calcium sediment as the specific marker for osteogenic maturation. At first, conditioned media was discard completely and washed with phosphate buffer saline (PBS) and fixed with 4% paraformaldehyde. After that, alizarin red stain was added to flask for 10 minutes and extra alizarin red was washed with PBS. The cells that had red color under microscope (calcium sedimentation) were positive for osteoblast differentiation since calcium sedimentation is a marker for osteoblast maturation (21).

## Results

### Expression of sclerostin by human myeloma cell lines, and T293 and K562

**Fig 1:**
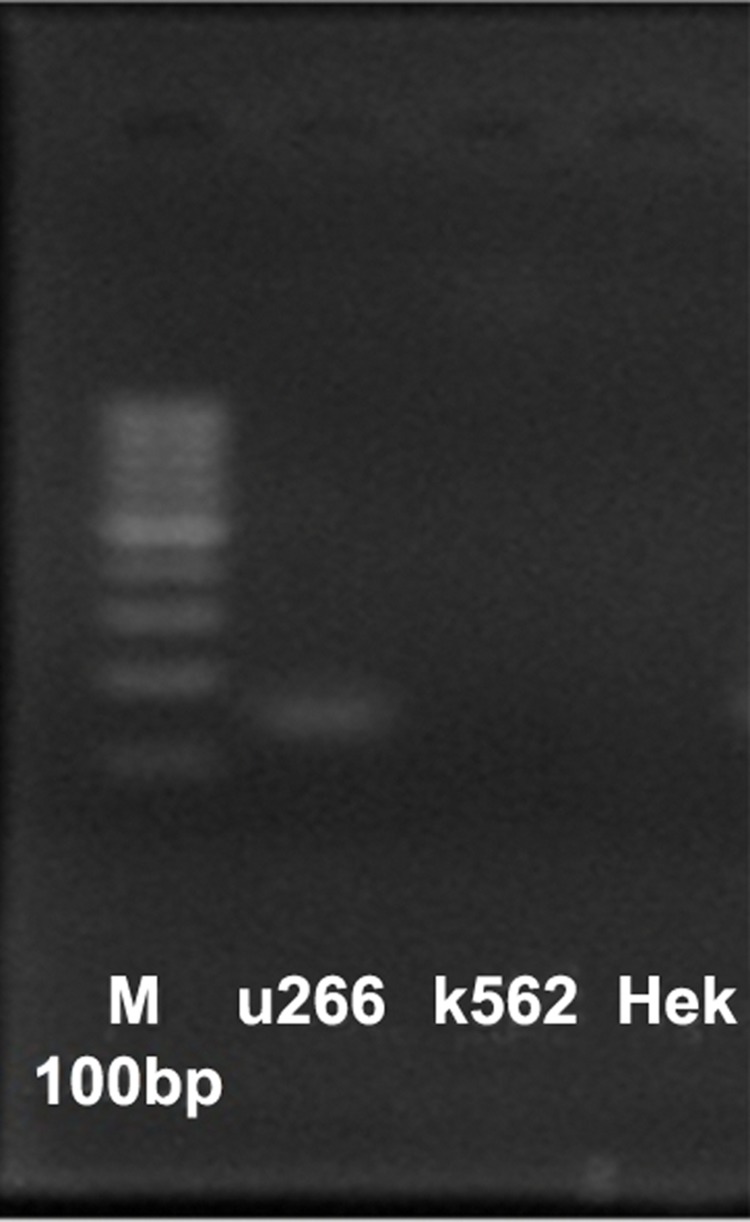
T in U266 cell line (myeloma), K562 and T293, U266 cell line has significant *SOST* expres sion but K562 and T293 do not.

**Fig 2:**
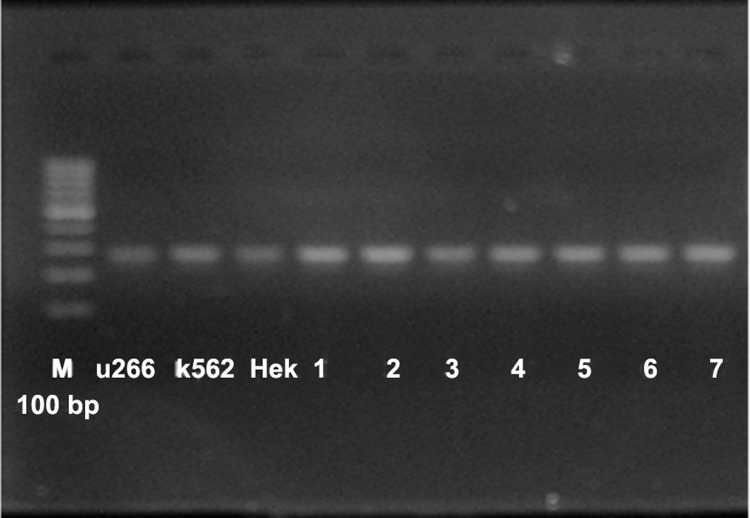
Evaluation of β2microglobin: all of samples expressed β2microglobin and we could confirm all of samples have good quality cDNA.

Expression of sclerostin in human myeloma cell line (U266) was evaluated as myeloma cells with myeloma markers (CD38+ , CD138+ , CD19- ) and T293 and K562 as negative control. Also sclerostin was evaluated in plasma cells from bone marrow of MM patients that expressed CD138. After RNA extraction, RT-PCR was done on these cells and we detected that *SOST* has good expression in U266 but not significantly in T293 and K562 (Fig 1). Also for assurance that all of cDNA are reliable we evaluated β2microglobin expression where it was present in all cDNA samples (Fig 2).

### Evaluation of *SOST* expression in plasma cells from MM patients

**Fig 3:**
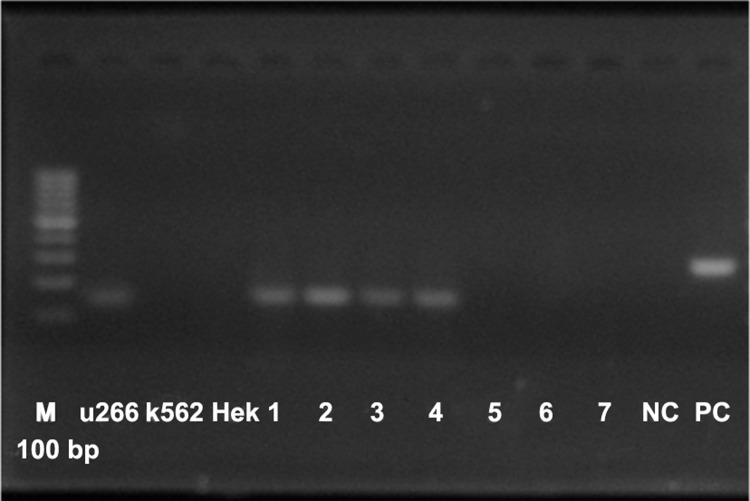
Evaluation of *SOST* expression in all of samples: 1, 2, 3, 4: Patients that their BM had more than 70% Plasma cells. 5: A patient that doesn’t have MM. 6: A patient with MM that got Talidomide for 2 years and her BM had 5% plasma cell.7: Patient with MM that got BMT. NC: Negative control. PC: Positive control.

Results show all of the plasma cells significantly express *SOST* and yet do not have any report for *SOST* in plasma cells from MM patients whether it is during transcription (mRNA) and or in protein expression. Also the patients that got treatment with Talidomide or BMT (bone marrow transplantation) don’t have significant expression of *SOST* (Fig 3).

### Evaluation of RUNX2 as a osteoblast marker after osteoblast differentiation in osteogenic medium on UCSC

**Fig 4:**
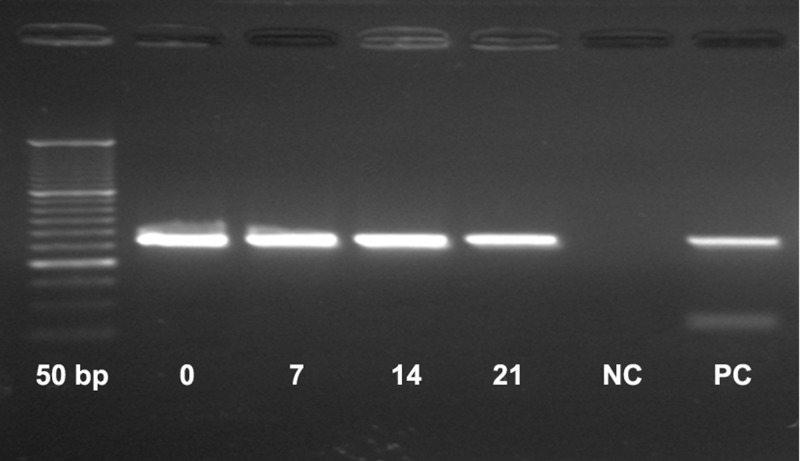
Result of Runx2 expression from culture UCSC in osteogenic medium for osteoblast differentiation: 0, 7, 14, 21 days after UCSC culture in osteogenic medium consisting of U266 conditioned media.

It is known that sclerostin is a secretory protein then should be secreted into conditioned media of MM cell line (U266) and when we add this condition media to osteogenic medium consisting of UCSC, it inhibits osteoblast differentiation and RUNX2 as essential marker for osteoblast lineage is not expressed because Runx2 is an essential factor for expression of collagen type I, Osteopontin and bone sialoprotein (9, 22). Therefore we could not detect RUNX2 expression after 21 days culture UCSC in osteogenic medium consisting of myeloma cell line condition media (Fig 4).

### Alizarin red stain for confirmation of calcium sedimentation

**Fig 5:**
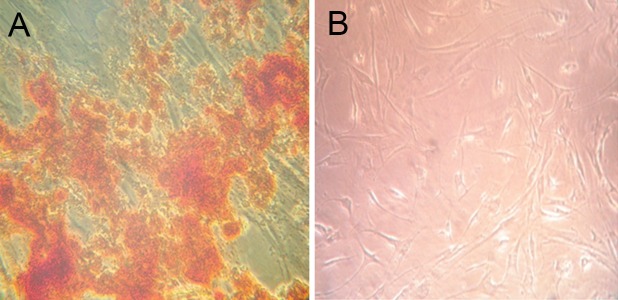
Result of alizarin red staining on UCSC after osteoblast differentiation without U266 conditioned media to confirm Runx2 expression. A: first day (Negative alizarin red). B: 21th day (Positive alizarin red).

Alizarin red staining was used to confirm calcium sedimentation as a result of osteogenic differentiation therefore at the 21th day the cells that were alizarin red positive showed calcium sedimentation and verified osteogenic differentiation. Our result show that after the 21th day UCSC in osteogenic medium without condition media of myeloma cell lines have alizarin red positive but UCSC in osteogenic medium with condition media of myeloma cell lines were not positive for alizarin red after 21 days (Fig 5). 

## Discussion

Bone disease is one of the most frequent problems in MM patients and it has been demonstrated that in MM patients, bone does not have a balance between bone formation (Osteoblastogenesis) and bone resorption (Osteoclasto genesis) (6, 23). In this study, we focused on the Wnt pathway because it has been demonstrated that the Wnt pathway induce bone formation via increase of expression of osteoblast transcription factors such as β-catenin and inhibit bone resorption via reducing the RANKL/OPG ratio (11, 24, 25). Therefore induction of Wnt signaling with a specific factor will have two advantages: first, inducing bone formation via increase of osteoblast cells and second, inhibit bone resorption via reduction of osteoclast cells. Among osteoblast inhibiting agents like DKK, Sfrp and sclerostin, sclerostin has the highest specificity. In addition, Wnt signaling exists in many different cells and inhibition or induction of Wnt signaling with nonspecific agents will be accompanied with different side effects but Sclerostin specifically inhibits Wnt signaling in osteoblast Therefore inhibition of *SOST*, induces bone formation in MM patients with the least side effect (26). There are good examples of *SOST* inhibition in diseases such as Van Buchem and Sclerosteosis, where both diseases, despite an increase of Wnt signaling activity, increase bone mass with no other problems or tumor (27). Also Gaur et al. have demonstrated that Wnt signaling induces bone formation directly by stimulating Runx2 expression as an essential transcription factor for osteoblast maturation (11, 25). Colucci et al. also studied MM patients and verified that plasma cells of MM patients have significant expression of *SOST* (26).

## Conclusion

In this study, we showed that *SOST* was expressed in plasma cells from MM patients. We also detected myeloma cells inhibiting Runx2 expression as an essential transcription factor for osteoblast maturation. That is why we propose that the inhibition of *SOST* may solve bone disease with the least side effect in MM patients. On the other hand, MM patients are usually old (usually≥50 years) and cannot suffer treatments such as radiotherapy and chemotherapy so it is reasonable that we treat bone disease with noninvasive procedures. We propose that inhibition of Sclerostin with antibody or alternative methods may prevent bone lesion in MM patients with the best result (without or at least side effect). However, it is strongly recommended that this antibody or other alternative procedure like epigenetic procedures are first done on animal models before on MM patients.
